# Relationship Between Big Five Personality Dimensions, Chronotype, and DSM-V Personality Disorders

**DOI:** 10.3389/fnetp.2021.729113

**Published:** 2021-08-23

**Authors:** Naomi Staller, Christoph Randler

**Affiliations:** Eberhard Karls University Tübingen, Tübingen, Germany

**Keywords:** morningness-eveningness, personality disorders, messi, big five, PID-5

## Abstract

Morningness-eveningness (M/E) is an important variable in individual differences and has an impact on many areas of life including general and mental health. In previous work eveningness has shown to correlate to personality disorders (PDs) and mental instability such as psychoticism, depression, and bipolar disorders. Therefore, a relationship between M/E and PDs can be assumed but has never been tested. The aim of this study was to assess a possible relationship between DSM-5-PDs and circadian timing (chronotype; M/E). We used the Morningness-Eveningness Stability Scale improved and clock time-based measurements, the PID-5 brief version, and the Big Five brief version. Sample: N = 630; mean age: 27.76 years, SD: 11.36 years; 137 male, 489 female, 4 diverse. In this short screening a relationship between eveningness and DSM-5-personality traits, (evening-oriented participants showing a higher PID-5 score: morningness -0.208/*p* < 0.001; eveningness: 0.153/*p* < 0.001) was found. Moreover, participants with high levels of distinctness (fluctuations of the perceived energy level during the day) are prone to PDs too, with distinctness being the best predictor for a high PID-5 score in this sample (0.299/*p* < 0.001). In the regression analysis, neuroticism, agreeableness, conscientiousness, and extraversion contributed significantly to the model with higher scores on extraversion, agreeableness and conscientiousness being related to lower scores on the PID-5. Neuroticism was positively related to PID-5 scores. Later midpoint of sleep (higher eveningness) was associated with higher PID-5 scores, as were higher fluctuations/amplitude during the day.

## Introduction

Human sleep behaviour differs considerably in terms of timing and preference and is referred to under the designation of ‘circadian rhythm’ (see e.g., Roenneberg, 2015). One facet of the circadian rhythm is ‘morningness-eveningness’ (M/E). Depending on the M/E, people tend to be awake, active, and able to solve cognitively demanding tasks early in the morning/late in the evening. Morningness-eveningness and chronotype are slightly separate constructs but are highly correlated with each other (r = 0.73; Zavada et al., 2005). While M/E expresses daytime preferences (e.g., preferred time for a workout), the chronotype describes actual behaviour associated with sleep habits (e.g., reduction in activity prior to sleeping) (Bauducco et al., 2020). Morning- and evening-types are the extrema of a continuum (both when M/E and chronotype is concerned) in which most people are categorized as neither-type with a tendency to one or the other extreme. As complement to M/E, a new facet was proposed describing variations of mood and performance throughout the day. The so called ‘distinctness’ (DI; or: amplitude of diurnal variation) facet reflects the change in the distribution of a person’s perceived energy level during the day. According to this description, a person’s circadian rhythm is composed of the behaviour associated with sleep habits (chronotype), the daytime preferences for demanding tasks (M/E) and the fluctuation of the perceived energy level during the day, concentration, and comprehension (distinctness).

Over the past decades the circadian typology received much attention in research because it is an interesting facet of individual differences (review: Adan et al., 2012). It was reported to correlate to various personality traits like conscientiousness ((+) to morningness, e.g., Randler, 2008; Tonetti et al., 2009; Tsaousis, 2010; Staller and Randler, 2021a) and openness ((+) to eveningness, e.g., Tsaousis, 2010; Randler et al., 2017a; Staller and Randler, 2021a; conflicting results: e.g., Randler, 2008; Tonetti et al., 2009). These relationships result in dependencies in many areas of life. For example, M/E shows effects on motivational regulation (e.g., morningness (+)/eveningness (-) to self-efficacy; Staller et al., 2021) or performance components (e.g., morningness (+)/eveningness (-) to *Organizational Citizenship Behavior*; Staller and Randler, 2021a). Risk-taking behaviour and personality traits summarized in the dark-triad also link to eveningness (Maestripieri, 2014; Marvel-Coen et al., 2018; Rahafar et al., 2017). Morning-types differ from evening-types even in their way of thinking and their creativity with evening-types relating more to creative thinking and being prone to ‘creative’ occupations (Caci et al., 2004; Díaz-Morales, 2007; Giampietro and Cavallera, 2007; for more physiological explanations see e.g., Facer-Childs et al., 2019). Also, evening-types report higher success in mate choice (Staller and Randler, 2021b) while morning-types show higher reproductive success in terms of number of children (Kasaeian et al., 2019; Staller and Randler, 2021b). The circadian typology repeatedly proved to be a strong predictor when examining differences in personality. In addition to the predictive nature of individual differences, it becomes apparent that it also relates to many aspects of PDs (Lyall et al., 2018).

When PDs are concerned, studies using unidimensional measures examining the M/E often show a relationship of eveningness to negative emotionality. For example, evening-types are more prone to developing seasonal affective disorder (Tonetti et al., 2012). Furthermore, eveningness has been shown to correlate with negative affect (e.g., Carciofo, 2020a), higher stress levels (e.g., Roeser et al., 2012; perceived stress: You et al., 2020), and tendencies to resort to external stimuli (caffeine (Fleig and Randler, 2009), smoking/soft drinks (Gariépy et al., 2019), excessive use of mobile phones (Demirhan et al., 2016; Randler et al., 2016), or long screen times (Gariépy et al., 2019; Shimura et al., 2018). Moreover, eveningness relates to anxiety (e.g., Cox and Olatunji, 2019) and depression (e.g., Au and Reece, 2017; Hasler et al., 2010; Randler et al., 2012), bipolar disorders (Ahn et al., 2008; Giglio et al., 2010) and antisocial behaviour in adolescents (Schlarb et al., 2014). Dong et al. (2019) reported adolescents prone to eveningness show poorer sleep health and therefore an increased risk of delayed emotional and cognitive development, anxiety disorders, depression, and physical symptoms (e.g., obesity). Together with circadian misalignment, these strengthen developmental tendencies towards impulsiveness and receptivity to reward systems, leading to risk-taking behaviour and increased likelihood of drug use and abuse (Logan et al., 2018).

Although, studies considering more dimensional measurements (M/E and distinctness) suggest low morning affect (MA) and high distinctness scores (DI) might be related to negative emotionality rather than the M/E preference itself (Konttinen et al., 2014; Jankowski 2016; Putilov, 2018; Carciofo, 2020b). MA relates to dimensions of the Big Five which are shown to have a positive effect on psychological wellbeing. So, high MA scores correlate to high conscientiousness and low neuroticism which in turn relate to life satisfaction (Drezno et al., 2019). Low conscientiousness and high neuroticism on the other hand correlate to depression, anxiety (Kotov et al., 2010), and poor sleep quality (Duggan et al., 2014). As for high DI, recent studies showed a connection to neuroticism and poor sleep quality (Carciofo and Song, 2019; Carciofo 2020b). Moreover, it shows a relationship to negative effect (Carciofo and Song, 2019), and weak stress-coping-strategies (Oginska and Oginska-Bruchal, 2014) and higher perceived stress levels (You et al., 2020).

These studies point out a relationship of M/E (eveningness/low morning affect) and distinctness to personality disorders (PDs) and mental instability. The degree to which a trait is considered a personality characteristic is in some cases not clearly distinguishable from a PD. To clarify the relations, the five-factor model (Big Five: neuroticism, extraversion, conscientiousness, agreeableness, openness) and the three-factor model of personality (Big Three: negative emotionality, positive emotionality, and disinhibition versus constraint) have been studied in personality psychology extensively. In a quantitative review Kotov et al. (2010) reported that neuroticism was the strongest predictor of PDs followed by low conscientiousness. Low Extraversion was also a good predictor for many disorders with highest effect sizes for dysthymic disorder and social phobia (Kotov et al., 2010). Disinhibition as one of the Big Three was linked to few disorders, e.g., substance use disorders, openness and agreeableness showed almost no predictive value (Kotov et al., 2010). Kotov et al. (2010) showed a strong relation of ‘common mental disorders’ to personality dimensions with Big Five being better predictor dimensions than Big Three.

### Study Objectives

The aim of this study was to assess the relationship between PDs listed in the Diagnostic and statistical manual of mental disorders 5th edition (American Psychiatric Association, 2013a) and circadian timing. We measured circadian timing by a preference scale and clock-based measurements to further assess sleep duration and midpoint of sleep. We expected a positive relationship between eveningness/low morning affect, and distinctness with DSM-5-personality traits.

## Methods

### Participants and Data Collection

This study was carried out in accordance with the Declaration of Helsinki for experiments involving humans. Participants (*N* = 630) mean age and gender was 27.76 years (SD: 11.36 years); 137 males (mean age: 30.31; SD: 13.48), 489 females (mean age: 27.08; SD: 10.61), 4 diverse (diverse refers to all participants who do not identify as male or female; mean age: 23.33; SD: 8.39). Diverse participants were included in all correlational analysis but dropped from the gender analysis because of the low sample size. Data was anonymously collected using an online survey hosted on the platform SoSciSurvey. We collected data from October 29, 2020 until November 29, 2020, after daylight saving time and in a short study period of 1 month. Therefore, seasonal and solar effects are neglectable. Participants were made aware of the study via different social media platforms and mail distribution lists (e.g., University-mailing lists which reached students and employees alike). We informed participants about the voluntariness, the option to stop the questionnaire at any point and ensured formal consent.

### Statistical Analysis

For the statistical analysis we used SPSS version 27.0. We first calculated Pearson’s correlations of the relationship between the personality and sleep variables and the relationship between PID-5 (full score/sub-scales) and the predictors Big Five and sleep variables followed by a linear regression analysis. We used parametric testing and followed the central limit theorem (CLT) that states that the distribution of sample means approximates a normal distribution as the sample size gets larger, as a rule of thumb, samples exceeding N = 500 can be used in parametric testing. Kolmogorov-Smirnov tests showed a deviance from normal distribution (*p* < 0.01 in all variables), while the Q-Q plots from these variables suggested a normal distribution. The standardized residuals of the PID-V score from the multiple linear regressions were normal distributed.

### Morningness-Eveningness Stability Scale (MESSi)

Morningness-eveningness (M/E) is based on an intrinsic (endogenous) rhythm of the human body which can be assessed using different methods (e.g., actigraphy/questionnaires/dim-light melatonin onset/or blood sample assays (BodyTime; Wittenbrink et al., 2018)). We here used the Morningness-Eveningness-Stability-Scale improved (MESSi; Randler et al., 2016; Díaz-Morales et al., 2017) together with the corrected midpoint of sleep to determine participants’ circadian timing. The MESSi examines three different scales influencing the M/E - the morning affect (MA, e.g.: How alert do you feel during the first half hour after having awakened in the morning?), the eveningness subscale (EV, e.g.: In general, how is your energy level in the evening?) and the distinctness subscale (DI, e.g.: There are moments during the day where I feel unable to do anything. response options: ‘totally’ to ‘not at all’). Each scale is composed of five items in a 1-5 Likert-format. Higher scores indicate higher morningness (MA), eveningness (EV) or daytime fluctuations of the subjective felt energy level during the day (DI). Factorial invariance, structure and reliability were confirmed using different models and languages (Díaz-Morales and Randler, 2017; Rahafar et al., 2017; Randler et al., 2016; Rodrigues et al., 2018; Tomažič and Randler, 2020; Faßl et al., 2019). In the current sample the Cronbach’s α were 0.910 for MA, 0.893 for EV, and 0.772 for DI.

### Habitual Sleep-Wake Variables

Habitual sleep-wake variables were assessed to calculate different measures: sleep duration, midpoint of sleep and social jetlag. We asked participants for bedtimes and time of rising on weekdays/workdays and weekend days/free days. Sleep duration (SD) was calculated from the difference between bedtime and risetime. Average sleep duration was summed up as follows:

$$SD_{average}=\frac{5xSD_{workdays}+2xSD_{freedays}}{7}$$

Midpoint of sleep (MS) is the midpoint between sleep onset and awakening in clock time. People sleep longer at the weekends compared to weekdays to compensate social jetlag. Social jetlag is defined as the difference between midpoint of sleep on free days and midpoint of sleep on weekdays (Wittmann et al., 2006). We therefore applied an algorithm to correct the weekend oversleep. This algorithm was proposed by Roenneberg et al. (2004):

$$MS_{freedays\;corrected}=\;MS_{freedays}-0,5x\left(\frac{SD_{freedays}-\;\left(5xSD_{workdays}+2xSD_{freedays}\right)}{7}\right)$$

### Personality Inventory for DSM-5—Brief Form (PID-5-BF)

The Personality Inventory for DSM-5 questionnaire (PID-5; American Psychiatric Association, 2013b) was used in its brief German form containing 25 items related to PDs (Krueger et al., 2015). This questionnaire represents a short screening, not a detailed analysis. We chose this instrument because it gives a broad overview over anomalies in personality and PDs. Each item on the measure is rated on a 4-point scale (i.e., 0 = very false or often false; 1 = sometimes or somewhat false; 2 = sometimes or somewhat true; 3 = very true or often true). Additionally, we calculated the five subdomains, each consisting of five items: negative affect, detachment, antagonism, disinhibition, and psychoticism.

### Big Five Inventory-10

We used a short version of the Big Five Inventory (BFI-10: Rammstedt and John, 2007). This scale consists of 10 items with two items for each personality dimension (Extraversion, Agreeableness, Openness, Neuroticism, Conscientiousness) and is rated in a five-point Likert format. One item per dimension is reverse coded. This version of the Big Five questionnaire was developed to provide a measurement which retains significant levels of reliability and validity although, drastically reducing the response time. The BFI-10 shows acceptable psychometric properties given its brevity and is suggested for research settings with very limited participant time (Rammstedt and John, 2007). Since this study was designed as a screening study to investigate a relationship between the chronotype and PDs, we chose this version to gain further insight into associations with personality without overstressing the overall length of the questionnaire.

## Results

Descriptive statistics of the sample are presented in Table 1. We could confirm the positive relationship of morningness as well as the negative relationship of eveningness with conscientiousness (Table 2). Moreover, eveningness correlated to openness. These results are further strengthened by the analysis of the relationships of midpoint of sleep with conscientiousness (negative relationship ≙ −) and openness (positive relationship ≙ +), meaning that late sleepers are less conscientious and more open. In this sample morningness was correlated with extraversion (+). Distinctness correlated to neuroticism (+), extraversion (−) and conscientiousness (−).

**TABLE 1:** Descriptive statistics of the sample (*N* = 630: 489 female, 137 male, 4 diverse).

	Mean	Standard deviation
Midpoint of sleep (corrected) Unit of measurement: clock time	04:43	1:30
Morning affect	2.91	0.99
Eveningness	3.30	0.99
Distinctness	3.56	0.77
Extraversion	3.02	1.10
Agreeableness	3.26	0.79
Conscientiousness	3.54	0.85
Neuroticism	3.24	1.03
Openness	3.73	1.02
Negative Affectivity	1.18	0.59
Reticence	0.90	0.58
Antagonism	0.42	0.44
Disinhibition	0.65	0.52
Psychoticism	0.90	0.64
PID-5	4.05	1.86
Age (years)	27.76	11.36

**TABLE 2:** Relationship between personality and sleep variables.

		Extraversion	Agreeableness	Conscientiousness	Neuroticism	Openness
Morning affect	Pearson’s correlations	0.088^*^	0.061	0.344^**^	−0.144^**^	−0.047
		*p*-value	0.027	0.129	<0.001	<0.001	0.240
Eveningness	Pearson’s correlations	0.004	−0.050	−0.235^**^	−0.042	0.155^**^
		*p*-value	0.927	0.211	<0.001	0.288	<0.001
Distinctness	Pearson’s correlations	−0.141^**^	−0.033	−0.270^**^	0.244^**^	0.036
		*p*-value	<0.001	0.407	<0.001	<0.001	0.371
Midpoint of sleep (corrected). Unit of measurement: clock time	Pearson’s correlations	−0.011	−0.049	−0.274^**^	−0.034	0.113^**^
		*p*-value	0.787	0.224	<0.001	0.398	0.005
Average sleep duration	Pearson’s correlations	−0.074	−0.024	−0.067	−0.008	0.035
		*p*-value	0.066	0.548	0.095	0.845	0.379

*p < 0.05; **p < 0.01.

Morningness correlated negatively with negative affectivity, reticence, disinhibition, psychoticism, and the PID-5 score, while eveningness correlated positively to reticence, disinhibition, psychoticism, and the PID-5 score (Table 3). Moreover, distinctness correlated to negative affectivity (+), reticence (+), disinhibition (+), psychoticism (+), and the PID-5 score (+). Like the results of eveningness, midpoint of sleep was positively correlated to reticence, antagonism, disinhibition, psychoticism, and the PID-5 score. Late chronotypes related to the PID-5 scales and subdomains irrelevant of the data collection method, but the correlation of midpoint of sleep with PID-5 was a little higher than the relationship of eveningness (+) or morning affect (-). Nevertheless, PDs are related to eveningness (Table 3). Conscientiousness related negatively to all PID-5 scales and subdomains, while neuroticism related positively to all but antagonism. Agreeableness correlated negatively to negative affectivity, reticence, antagonism, and the PID-5 score. Extraversion related negatively to negative affectivity, reticence, psychoticism, and the PID-5 score but positively to antagonism. Openness showed to be the weakest predictor with a negative relationship to reticence and a positive to psychoticism (Table 3).

**TABLE 3:** Relationship between PID-5 (full score and sub-scales) and the predictors Big Five and sleep variables.

		Negative affectivity	Reticence	Antagonism	Disinhibition	Psychoticism	PID-5
Morning affect	Pearson’s correlations	−0.176^**^	−0.169^**^	0.006	−0.131^**^	−0.190^**^	−0.208^**^
		*p*-value	<0.001	<0.001	−0.873	0.001	<0.001	<0.001
Eveningness	Pearson’s correlations	0.030	0.110^**^	0.065	0.135^**^	0.165^**^	0.153^**^
		*p*-value	0.447	−0.006	0.102	0.001	<0.001	<0.001
Distinctness	Pearson’s correlations	0.309^**^	0.206^**^	0.046	0.142^**^	0.253^**^	0.299^**^
		*p*-value	<0.001	<0.001	−0.254	<0.001	<0.001	<0.001
Midpoint of sleep (corrected). Unit of measurement: clock time	Pearson’s correlations	0.075	0.128^**^	0.105^**^	0.173^**^	0.220^**^	211^**^
		*p*-value	0.061	−0.001	0.009	<0.001	<0.001	<0.001
Average sleep duration. Unit of measurement: hours	Pearson’s correlations	0.055	−0.011	−0.030	−0.031	−0.011	−0.006
		*p*-value	0.169	0.775	0.456	0.445	0.776	0.889
Extraversion	Pearson’s correlations	−0.180^**^	−0.481^**^	0.130^**^	0.034	−0.197^**^	−0.233^**^
		*p*-value	<0.001	<0.001	−0.001	0.388	<0.001	<0.001
Agreeableness	Pearson’s correlations	−0.098^*^	−0.231^**^	−0.295^**^	−0.071	−0.007	−0.194^**^
		*p*-value	0.014	<0.001	<0.001	0.074	0.856	<0.001
Conscientiousness	Pearson’s correlations	−0.198^**^	−0.244^**^	−0.166^**^	−0.372^**^	−0.252^**^	−0.368^**^
		*p*-value	<0.001	<0.001	<0.001	<0.001	<0.001	<0.001
Neuroticism	Pearson’s correlations	0.570^**^	0.243^**^	0.008	0.159^**^	0.237^**^	0.383^**^
		*p*-value	<0.001	<0.001	0.851	<0.001	0.001	<0.001
Openness	Pearson’s correlations	0.026	−0.095^*^	−0.018	0.035	0.189^**^	0.049
		*p*-value	0.521	0.017	0.659	0.386	<0.001	0.223

*p < 0.05; **p < 0.01.

To assess the influence of all variables simultaneously, we used a hierarchical regression. In step 1, age and gender were included (*R* ^2^ = 0.037; change in *R* ^2^ = 0.037), in step 2 personality (*R* ^2^ = 0.333; change in *R* ^2^ = 0.296), and in step 3 sleep variables (*R* ^2^ = 0.359; change in *R* ^2^ = 0.026). In the final regression model, we used sleep variables, Big Five dimensions and the demographics age and gender as predictor variables while the PID-5 total score acted as dependent variable. The full model was highly significant (F_12.612_ = 28.565, *p* < 0.001; with an *R* ^2^ = 0.36). Collinearity was negligible with a maximum VIF in one variable of 2.0. Concerning the personality dimensions, neuroticism, agreeableness, conscientiousness, and extraversion contributed significantly to the model (Table 4) with higher scores on extraversion, agreeableness and conscientiousness being related to lower scores on the PID-5. Neuroticism was positively related to PID-5 scores. Concerning sleep variables, later midpoint of sleep (higher eveningness) was associated with higher PID-5 scores, as were higher fluctuations/amplitude during the day. Older age and being male were associated with lower scores on the PID-5 (Table 4).

**TABLE 4:** Results of the linear regression model with the PID-5 score as dependent variable.

	Beta	T	sig
Constant		1.870	0.062
Average sleep duration. Unit of measurement: hours	−0.056	−1.650	0.099
Midpoint of sleep (corrected). Unit of measurement: clock time	0.094	2.193	0.029
Morning affect	0.066	1.453	0.147
Eveningness	0.040	0.856	0.393
Distinctness	0.136	3.759**	<0.001
Extraversion	−0.109	−3.173**	0.002
Agreeableness	−0.165	−5.051**	<0.001
Conscientiousness	−0.271	−7.360**	<0.001
Neuroticism	0.334	9.202**	<0.001
Openness	0.052	1.542	0.124
Age	−0.088	−2.444*	0.015
Unit of measurement: years Gender (male (1)/female (2)	−0.079	−2.253*	0.025

*p < 0.05; **p < 0.01.

## Discussion

Our results suggest that evening-types are prone to PDs regardless of the construct used (MESSi/midpoint of sleep). With morningness correlating negatively and eveningness correlating positively with PID-5 risk the chronotype depicted as a continuum also maps a continuous relationship to PID-5 risk. Morningness hereby acts as one extreme, with low PID-5 risk while eveningness puts the other extreme with high PID-5 risk. We therefore could confirm the hypothesis that eveningness is related to high risk of PDs. The correlation of midpoint of sleep with the PID-5 score was higher than the correlation of eveningness. This might be since midpoint of sleep is a variable of the chronotype (biological) while eveningness is a facet of M/E (psychological). The chronotype and M/E are separate constructs. While the chronotype is more concerned with actual measurable behaviour, M/E assesses subjectively perceived preferences (Bauducco et al., 2020). This was corroborated by the analysis of multicollinearity.

Furthermore, the distinctness subscale is one of the best predictors of high PID-5 risk, showing more predictive power than morningness/eveningness or any other sleep related variable in this sample. The reason for this might be the perceived pressure of societal expectations. At school or the workplace for example, it is expected, that students/employees perform their work and take their breaks at the assigned times. People with a strong fluctuating amplitude throughout the day are less able to meet this demand than those with less fluctuation. This could subsequently lead to feelings of inadequacy, which in the further course lay the foundation for PDs.

These results open venues for further research and show that circadian fluctuations of mood and performance may be predictors for emerging PDs. Furthermore, they strengthen previous work pointing out the importance of DI when emotional lability is considered (e.g., Ogińska et al., 2017; Carciofo, 2020b). We propose reconfirming the results by the PID-5 full version questionnaire. Moreover, to build on that small body of literature DI should be further assessed in combination with an objective chronotype measure, such as actigraphy (Faßl et al., 2019).

The analysis of the predictive relationship of the Big Five to the PID-5 criteria yielded similar results to those described earlier by Kotov et al. (2010). We here showed that the strongest predictor of low PID-5 risk (combined score/subdomains) is a high value in conscientiousness. In our sample agreeableness and extraversion were good predictors of low PID-5 risk, while Kotov et al. (2010) reported agreeableness to have almost no predictive power. A high value in neuroticism on the other hand was the best predictor for high PID-5 risk. Moreover, neuroticism was the value with the strongest predictive power on the PID-5 score. Here, openness had almost no predictive value, as previously found by Kotov et al. (2010) too.

There is a direct correlation between low extraversion, low conscientiousness and high neuroticism and increased risk of PID-5 in both the present sample and Kotov’s sample. The negative association between morningness and PID-5 risk may therefore be further strengthened at a secondary level by the fact that morningness is associated with high extraversion, high conscientiousness, and low neuroticism. Against this background, the positive association between eveningness and PID-5 risk is also further reinforced, since eveningness is correlated with low conscientiousness. Again, the distinctness subscale emerges as a stronger predictor of high PID-5 risk (at this secondary level) than eveningness, through its association with low extraversion, low conscientiousness, and high neuroticism. We therefore conclude that evening-types with high values in distinctness represent the high-risk group for PDs while morning-types with low values in distinctness have a rather low risk.

Furthermore, we replicated well known correlations of the chronotype. Morningness was positively while eveningness was negatively related to conscientiousness (e.g., Randler, 2008; Tonetti et al., 2009; Tsaousis, 2010; Staller and Randler, 2021a). Eveningness correlated to openness as previously shown by e.g., Tsaousis (2010); Randler et al. (2017b); Staller and Randler (2021a), for conflicting results see e.g., Randler (2008); Tonetti et al. (2009).

## Strengths and Limitations

This study presents an interesting investigation of the effect of chronotype on PID-5 risk. What is particularly interesting about this work is that it builds on a small, but growing, body of literature exploring the relationship between chronotype and PDs. We here showed a clear relationship of chronotype and PID-5 risk. Furthermore, through the choice of survey method, we were able to identify another strong predictor of high PID-5 risk–the distinctness scale of the MESSi. These results may mark the MESSi a valuable tool for the rapid screening of a large group for PID-5 risk. To confirm this scope, the construct should be tested with the actual diagnostic methods used in clinical psychology. A further limitation of this work besides the screening nature of the methods rather than an in-depth analysis is the study sample. It does not represent a cross-section of the population and consisted of significantly more women than men. We suggest repeating this study with a larger and more representative sample, and appropriate in-depth diagnostic tools to follow up on the evidence of the close relationship between morningness-eveningness/distinctness with the occurrence of PDs that we have uncovered through the present study. Another point may be the assessment of moderator variables. In the meta-analysis of Au and Reece (2017) potential moderator variables of the relationship between morningness-eveningness and mood symptom severity have been identified. Apart from the questionnaire used (MEQ vs CSM), no variables showed a significant outcome. The authors have tested gender, age groups, clinical versus non-clinical samples, different disorders.

Further, we did not adjust the calculation of the chronotype midpoint of sleep on free days algorithm. It would be interesting to individually modify this measure to take actual relation of workdays and work-free days into account, because the standard algorithm assumes 5 workdays and 2 free days (to calculate average sleep duration). However, in a previous study in another sample, we found no significant difference in chronotype during and before the pandemic (Staller and Randler, 2021b).

## Data Availability

The raw data supporting the conclusion of this article will be made available by the authors, without undue reservation.
